# Falcarindiol Isolated from *Notopterygium incisum* Inhibits the Quorum Sensing of *Pseudomonas aeruginosa*

**DOI:** 10.3390/molecules26195896

**Published:** 2021-09-29

**Authors:** Chaoyue Zhao, Hongda Zheng, Liman Zhou, Hongrui Ji, Lu Zhao, Wengong Yu, Qianhong Gong

**Affiliations:** 1 School of Medicine and Pharmacy, Ocean University of China, Yushan Road, Qingdao 266003, China; zhaochaoyue2712@stu.ouc.edu.cn (C.Z.); hongdazh@yeah.net (H.Z.); zhouliman88@126.com (L.Z.); jhr@stu.ouc.edu.cn (H.J.); zhaolu@stu.ouc.edu.cn (L.Z.); yuwg66@ouc.edu.cn (W.Y.); 2 Laboratory for Marine Drugs and Bioproducts, Qingdao National Laboratory for Marine Science and Technology, 1 Wenhai Road, Qingdao 266237, China; 3 Provincial Key Laboratory of Glycoscience and Glycotechnology, Ocean University of China, Yushan Road, Qingdao 266003, China; 4 Key Laboratory of Marine Drugs, Chinese Ministry of Education, School of Medicine and Pharmacy, Ocean University of China, 5 Yushan Road, Qingdao 266003, China

**Keywords:** falcarindiol, quorum sensing, *Pseudomonas aeruginosa*, *Notopterygium incisum*, LasR

## Abstract

Quorum sensing (QS) is employed by the opportunistic pathogen *Pseudomonas aeruginosa* to regulate physiological behaviors and virulence. QS inhibitors (QSIs) are potential anti-virulence agents for the therapy of *P. aeruginosa* infection. During the screening for QSIs from Chinese herbal medicines, falcarindiol (the major constituent of *Notopterygium incisum*) exhibited QS inhibitory activity. The subinhibitory concentration of falcarindiol exerted significant inhibitory effects on the formation of biofilm and the production of virulence factors such as elastase, pyocyanin, and rhamnolipid. The mRNA expression of QS-related genes (*lasB*, *phzH*, *rhlA*, *lasI*, *rhlI*, *pqsA*, and *rhlR*) was downregulated by falcarindiol while that of *lasR* was not affected by falcarindiol. The transcriptional activation of the *lasI* promoter was inhibited by falcarindiol in the *P. aeruginosa* QSIS-*lasI* selector. Further experiments confirmed that falcarindiol inhibited the *las* system using the reporter strain *Escherichia coli* MG4/pKDT17. Electrophoretic mobility shift assay (EMSA) showed that falcarindiol inhibited the binding of the transcription factor LasR and the *lasI* promoter region. Molecular docking showed that falcarindiol interacted with the Tyr47 residue, leading to LasR instability. The decrease of LasR-mediated transcriptional activation was responsible for the reduction of downstream gene expression, which further inhibited virulence production. The inhibition mechanism of falcarindiol to LasR provides a theoretical basis for its medicinal application.

## 1. Introduction

*Pseudomonas aeruginosa* is one of the most intractable opportunistic pathogens, and can cause acute and chronic human infections. Infections with *P. aeruginosa* are difficult to treat with antibiotics [1]. Antibiotics put enormous pressure on bacteria and lead to the emergence of multidrug-resistant strains [2]. Anti-virulence therapy is a novel anti-infection therapeutic strategy and could exert less selective pressure on bacteria [3]. Virulence in *P. aeruginosa* is mostly regulated by quorum sensing (QS), a complex network controlled by signal molecules. Therefore, quorum sensing inhibitors (QSIs) could be promising anti-virulence agents to treat *P. aeruginosa* infection [4].

QS regulates bacterial population behavior by secreting and detecting signal molecules. Three interwoven QS circuits are used by *P. aeruginosa*, including two acyl-homoserine lactone (AHL) systems (the *las* system and the *rhl* system) and the PqsR-controlled *pqs* system [5]. The *las* and *rhl* systems control the expression of more than 6% of the *P. aeruginosa* genome [6]. LasR works at the top of the regulatory hierarchy of the QS network [7,8]. LasI is the enzyme that synthesizes the signal molecular 3-oxo-dodecanoyl (3-oxo-C12)-HSL [9]. The receptor LasR binds with 3-oxo-C12-HSL and activates the expression of multiple genes, including *lasI*, *rhlI*, *pqsA*, *lasB*, *rhlR*, and *pqsR*, etc. [10]. Butyryl-HSL (C4-HSL) and alkyl quinolone signaling molecules activate and bind with the receptors RhlR and PqsR, respectively. RhlR inhibits the expression of *pqsR* and *pqsABCD* while PqsR activates that of *rhlRI*.

Inhibiting the QS response is one of the strategies plants have evolved to deal with bacterial infections [11]. Many natural extracts have inhibitory activities on the quorum sensing of *P. aeruginosa*. The extract of *Lavandula coronopifolia* can inhibit the biofilm formation of *P. aeruginosa*. Methanol extract from *Salix tetrasperma* stem bark can inhibit the swimming motility, swarming motility, and protease production of *P. aeruginosa* [12,13]. QSIs such as zingerone, baicalin, and naringenin have been found in natural extracts against the common pathogen *P. aeruginosa*, which can infect both humans and plants [14,15,16]. Our research identified the polyacetylenic compound falcarindiol from *Notopterygium incisum* as having QS inhibitory activity. The effect of falcarindiol on the virulence of *P. aeruginosa* and the mechanisms of anti-QS activity were further evaluated.

## 2. Results

### 2.1. Screening and Identification of Falcaridiol

**Figure 1:**
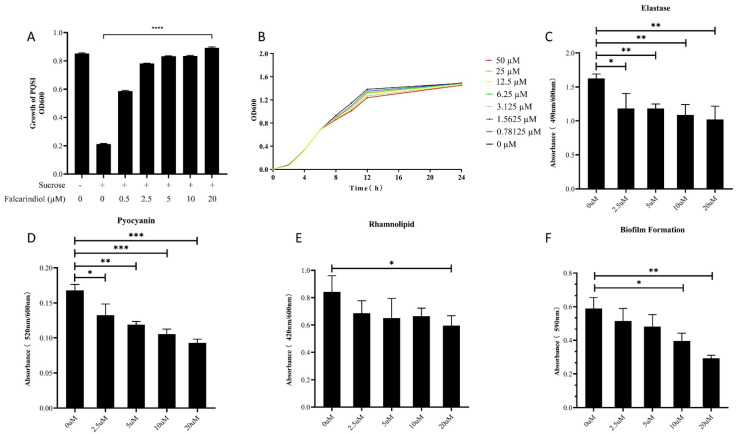
Effect of falcarindiol on PQSI selector (**A**), *P. aeruginosa* growth (**B**), elastase production (**C**), pyocyanin production (**D**), rhamnolipid production (**E**), and biofilm formation (**F**). All experiments were performed in triplicates. The data are shown as means ± standard errors. **** *p* < 0.0001, *** *p* < 0.001, ** *p* < 0.01, * *p* < 0.05.

To screen quorum-sensing inhibitors, we constructed the PQSI selector strain (*P. aeruginosa* PAO1 harboring a P*pqsA*-*sacB* plasmid). *SacB* is the sucrose lethal gene. The growth of the selector strain was inhibited in the presence of sucrose and PQS. QSIs rescued the bacteria by inhibiting the transcription of the *pqsA* promoter, and the OD600 nm value of the PQSI culture was increased. Because the *pqsA* promoter is activated by PqsR and LasR, the PQSI selector can detect QSIs which can inhibit either or both the *las* and *pqs* systems [17]. The *N. incisum* extract had the highest inhibitory activity against the PQSI selector in 10 herbal medicines (Figures S1 and S2). The purity of the active compound from the *N. incisum* extract was more than 98% by HPLC analysis (80% MeOH/H_2_O) (Figure S3). The active compound was identified as falcarindiol by using electrospray ionization mass spectrometry, nuclear magnetic resonance spectroscopy, and HPLC analysis (Figures S4–S6, Table S4). HPLC analysis showed that the proportion of falcarindiol in the *N. incisum* extract was about 30%. The effect of the chemical reference substance falcarindiol was detected by the PQSI selector. Falcarindiol inhibited the expression of *sacB* in the PQSI selector within a range of 0.5 μM to 20 μM (Figure 1A).

### 2.2. Effect of Falcarindiol on QS-Related Virulence Phenotype

In order to avoid imposing enormous selective pressure on bacteria, the subinhibitory concentrations of falcarindiol need to be identified. Growth curve analysis showed that the growth of PAO1 was unaffected by falcarindiol in the concentration range of 0.78125 µM to 50 µM (Figure 1B). The minimum inhibitory concentration (MIC) of falcarindiol to *P. aeruginosa* was greater than 400 µM (data not shown).

Elastase contributes to tissue injury and disrupts host immune responses during *P. aeruginosa* infection. Elastase is encoded by *lasB* and regulated by LasR [18]. Pyocyanin relates to diminished lung function in cystic fibrosis (CF) and promotes virulence due to its redox activity [19]. Rhamnolipid is a kind of biosurfactant regulated by the *rhlA*/*B* operon and causes the necrosis of polymorphonuclear leukocytes. The inhibitory effect of falcarindiol on virulence factors was dose-dependent, ranging from 2.5 µM to 20 µM. The production of elastase, pyocyanin, and rhamnolipid in PAO1 was inhibited by 37.17%, 44.71%, and 29.39% under 20 μM falcarindiol, respectively (Figure 1C–E). The swarming motility of *P. aeruginosa* is related to the production of rhamnolipid [20]. Correspondingly, the swarming motility of *P. aeruginosa* was also inhibited by falcarindiol (data not shown).

Bacteria attach to surfaces and are embedded in a protective extracellular matrix, such as extracellular polysaccharides and proteins, to form a biofilm. The formation of biofilm is closely associated with QS and related to the pathogenicity of *P. aeruginosa* [21]. Falcarindiol reduced biofilm formation in a dose-dependent manner, which was optimal at 20 μM, obtaining a 50.41% inhibition (Figure 1F).

### 2.3. Effect of Falcarindiol on Expression of QS-Regulated Genes

**Figure 2:**
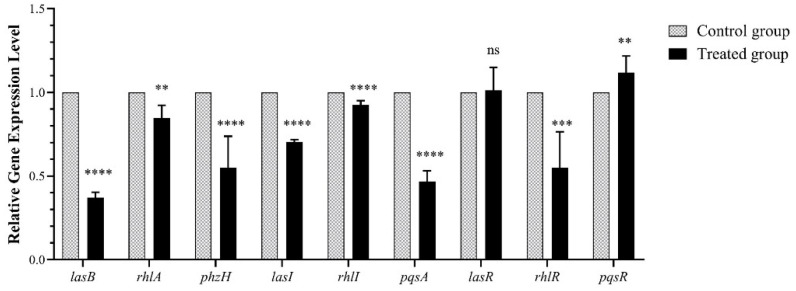
Effect of falcarindiol on mRNA expression. All experiments were performed in triplicates. The data are shown as means ± standard errors. **** *p* < 0.0001, *** *p* < 0.001, ** *p* < 0.01.

To investigate the effect of falcarindiol on the mRNA transcription of QS-related genes, *lasB*, *rhlA*, *phzH, lasI, rhlI, pqsA, lasR, rhlR*, and *pqsR* were selected for qRT-PCR detection. The virulence-related genes *lasB*, *rhlA*, and *phzH* were regulated by *las*, *rhl*, and *pqs* systems, respectively. After falcarindiol treatment, the mRNA expression of virulence-related genes *lasB*, *rhlA*, and *phzH* were downregulated by 63%, 22.98%, and 44.9%, respectively. *LasI*, *rhlI*, and *pqsABCD* direct the synthesis of signal molecules in *P. aeruginosa*. Falcarindiol reduced the mRNA expression of *lasI, rhlI*, and *pqsA* by 29.66%, 7.5%, and 53.3%, respectively. The mRNA expression of the QS receptor gene *rhlR* was reduced by 44.92%, that of *pqsR* was increased by 11.86%, and that of lasR was not affected by falcarindiol (Figure 2). The downregulation of *rhlI* and *rhlA* may be related to a decrease in the expression of *rhlR*. Because RhlR inhibits the expression of *pqsR* and *pqsABCD*, the upregulated expression of *pqsR* could be attributed to a decrease of *rhlR* expression [22]. The mRNA expression of *lasI*, *rhlI*, *pqsA*, and *rhlR* were downregulated, while the mRNA expression of their transcriptional activator LasR was not affected by falcarindiol. The downregulation of *lasI*, *rhlI*, *pqsA*, and *rhlR* may be caused by the inhibition of LasR-controlled transcriptional activation.

### 2.4. The Inhibition Effect of Falcarindiol on QS System

**Figure 3:**
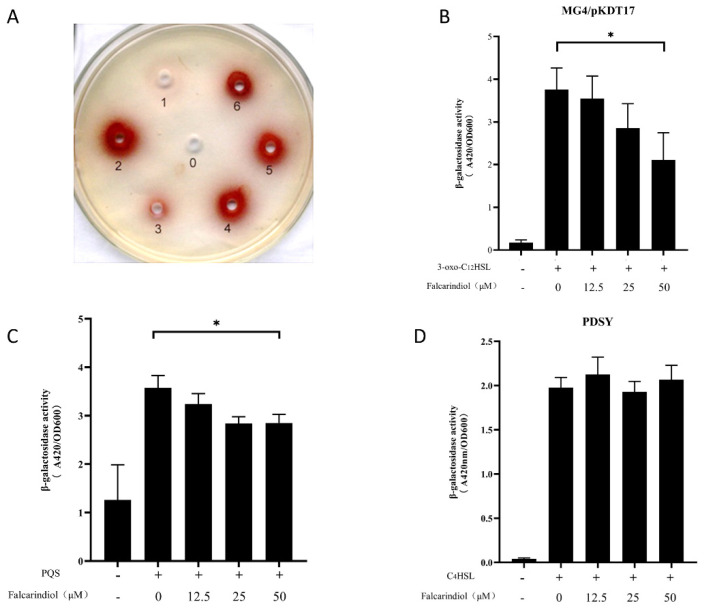
Effect of falcarindiol on QS inhibitory activity in *P. aeruginosa* QSIS-*lasI* (**A**). The negative control methanol was marked as 0. The positive control furanone C30 (5 mM) was marked as 1. The active ingredient (20 mM) from the *N. incisum* extract was marked as 2. Different concentrations of falcarindiol were marked as 3 to 6, and the concentrations were increased sequentially (5 mM, 10 mM, 20 mM, 50 mM). Effect of falcarindiol on *las* system in *E. coli* MG4/pKDT17 biosensor (**B**), *pqs* system in *E. coli* pEAL08−2 biosensor (**C**), and *rhl* system in *E. coli* pDSY biosensor (**D**). All experiments were performed in triplicates. The data are shown as means ± standard errors. * *p* < 0.05.

The *lasI* and *rhlI* double-mutant strain *P. aeruginosa* QSIS-*lasI* containing P*lasI*-*sacB* fusion and *lasR* gene was unable to survive due to the presence of 3-oxo-C12-HSL and sucrose. When QSIs were added to the medium, the QSIS-*lasI* selector strain was rescued, and a red zone was observed around the well. The rescued effect of falcarindiol on the QSIS-*lasI* selector strain was concentration-dependent, indicating that falcarindiol inhibited the transcriptional activation of the *lasI* promoter (Figure 3A). In order to avoid the interference of the *rhl* and *pqs* systems in *P. aeruginosa*, *E. coli* MG4/pKDT17 containing the *lasB*-*lacZ* fusion and *lasR* gene was used to investigate whether the transcriptional activation of LasR was inhibited by falcarindiol. *E. coli* pEAL08-2 and *E. coli* pDSY were used as the monitor strains for *pqs* and *rhl* systems, respectively. The inhibitory rate of falcarindiol (50 μM) on β-galactosidase activity reached 38.5%, indicating that falcarindiol could inhibit the transcription of LasR-controlled genes (Figure 3B). Falcarindiol also exhibited inhibitory activity in *E. coli* pEAL08-2 but not in *E. coli* pDSY (Figure 3C,D). Therefore, falcarindiol may play an inhibitory role in the *las* and *pqs* systems.

### 2.5. Effect of Falcarindiol on the Binding of LasR to the Promoter Regions

**Figure 4:**
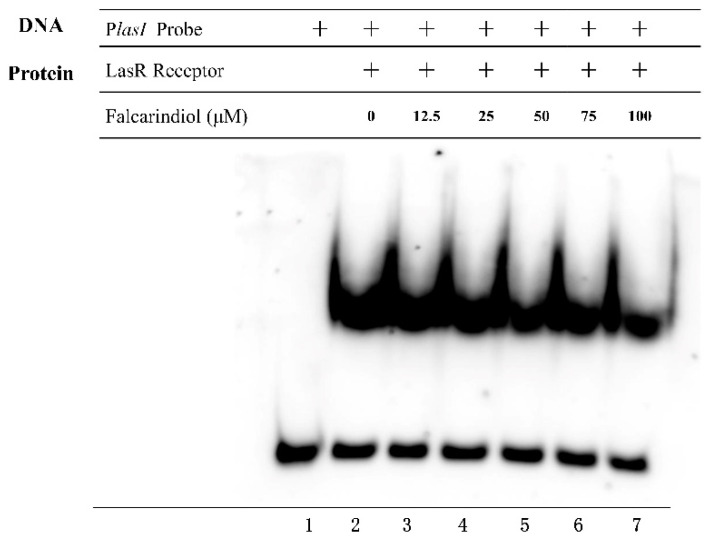
EMSA analysis of the effect of falcarindiol on the binding of LasR with promoter region of the *lasI* gene.

In order to detect whether falcarindiol could inhibit the binding of LasR with the promoters of downstream genes, we performed electrophoresis mobility shift assay (EMSA) experiments [23]. Falcarindiol interfered with the binding of LasR to the *lasI* promoter region (Figure 4). Falcarindiol inhibited the binding of LasR to the promoter of downstream genes, resulting in the inhibition of the expression of QS-regulated genes, which further affected the virulence traits of *P. aeruginosa*. The binding of PqsR with the *pqsA* promoter was not affected by falcarindiol (data not shown).

### 2.6. Molecular Docking Analysis

**Figure 5:**
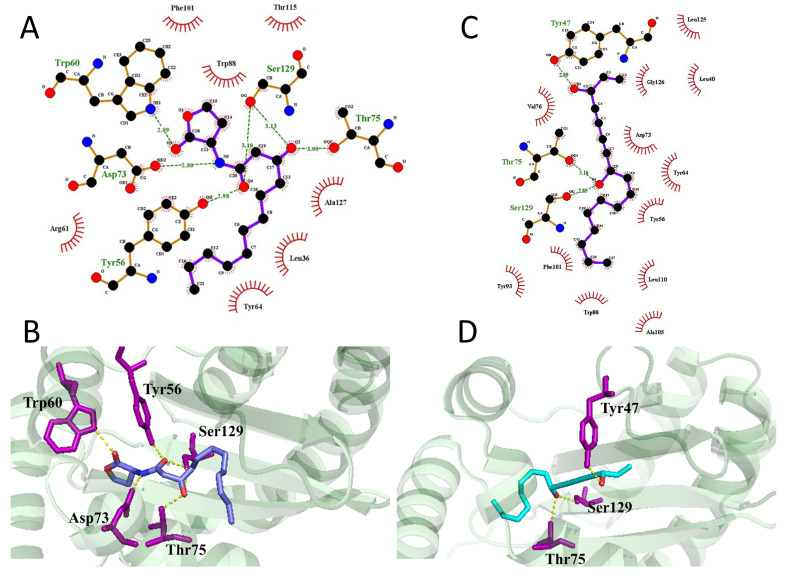
The 2D (**A**) and 3D (**B**) interaction maps of 3-oxo-C12-HSL and LasR. The 2D (**C**) and 3D (**D**) interaction maps of falcarindiol and LasR.

It is reported that 3-oxo-C12-HSL binds with LasR, which can then regulate the transcription of multiple downstream genes. Molecular docking was carried out to predict the binding of falcarindiol and LasR. The docking energies of LasR and falcarindiol (−7.5 kcal/mol) were similar to those of natural ligand and LasR (−8.0 kcal/mol) (Table S5). The natural ligand 3-oxo-C12-HSL was able to form five hydrogen bonds with amino acid residues (Tyr56, Trp60, Asp73, Thr75, Ser129) (Figure 5A,B). Falcarindiol was able to form three hydrogen bonds (Tyr47, Thr75, Ser129) (Figure 5C,D). In a manner similar to QSI butein, falcarindiol could interact with the Tyr47 residue and disrupt the stability of LasR [24]. The instability of LasR may inhibit binding with the promoter region of the target genes, in turn reducing its transcriptional activation.

## 3. Discussion

*P. aeruginosa* can easily acquire resistance to various antibiotics. QSIs can inhibit *P. aeruginosa* virulence without affecting bacterial growth, which imposes less selection pressure for bacterial resistance. Falcarindiol was identified from *N. incisum* and exhibited QS inhibitory activity. Our research provided a reference for the purification of the natural polyacetylenic compound. The research on the biological activity of falcarindiol provided a theoretical basis for the development and application of new antibiotics. Previous studies showed that falcarindiol had a variety of pharmacological activities, such as anti-apoptotic, antioxidant, and anti-inflammatory [25]. Falcarindiol also has anti-bacterial activity [26]. A study by Zhang et al. indicated that falcarindiol can inhibit the activation of *lasI*, *rhlI*, and *lasB* promoters in the *p-lux* promoter-reporter system [27]. However, the molecular mechanisms underlying the falcarindiol-mediated inhibition of QS remain unclear.

Using the *P. aeruginosa* QSIS-*lasI*, *E. coli* MG4/pKDT17, *E. coli* pEAL08-2, and *E. coli* pDSY assay, we found that falcarindiol could inhibit the *las* and *pqs* systems. Falcarindiol reduced the mRNA expression levels of *lasI*, *rhlI*, *pqsA*, and *rhlR*, which were activated by the transcriptional activator LasR. However, the mRNA expression of *lasR* was not affected by falcarindiol. EMSA results confirmed that falcarindiol inhibited the DNA-binding capability of LasR. Falcarindiol interfered with the transcriptional activity of LasR, resulting in a decreased expression of LasR-controlled downstream genes (*lasB*, *rhlA*, *phzH*, *lasI*, *rhlI*, *pqsA*, and *rhlR*). The inhibitory effect of falcarindiol on the *pqs* system may be controlled by other mechanisms. LasR bonded with 3-oxo-C12-HSL to activate transcription. Molecular docking results revealed that falcarindiol was able to form hydrogen bonds with Tyr47, destabilizing the conformation of LasR [28]. The binding of Tyr47 and ligands results in the ligand being unable to form hydrogen bonds with Trp60 and Asp73 residues [24]. Falcarindiol interfered with the binding of natural ligands with LasR. Meanwhile, the LasR-mediated transcriptional activation of downstream gene promoters was inhibited by falcarindiol in *P. aeruginosa* QSIS-*lasI* and *E. coli* MG4/pKDT17. Falcarindiol exhibited weak inhibitory activity against the AHL-mediated QS system in *Chromobacterium violaceum* (data not shown). Because of the long alkyl chain in its structure, falcarindiol showed a stronger inhibition effect on the *las* system than on the C6-HSL-mediated QS system. Therefore, falcarindiol was expected to be an inhibitor of the long-chain AHL-mediated QS system.

## 4. Materials and Methods

### 4.1. Bacterial Strains, Plasmids, and Materials

The bacterial strains and plasmids used in our research are listed in Table S1. The primers for PCR and real time RT-PCR are listed in Tables S2 and S3, respectively. The chemical reference substance falcarindiol was purchased from a Chengdu Purechem-Standard Co. (Chengdu, China), LTD, and was used to evaluate anti-QS activity against *P. aeruginosa* PAO1.

### 4.2. Screening of QSIs

The promoter region of *pqsA* was amplified and ligated into the *Sph*I and *Xba*I sites of plasmid P*lasB*-*sacB*1. The resultant recombinant plasmid P*pqsA*-*sacB* was transformed into *P. aeruginosa* PAO1 to construct the PQSI strain. The overnight culture of the PQSI strain was diluted 1:1000 in LB with 80 μg/mL gentamicin and 1.5% sucrose. Crude extracts (50 mg/mL) of Chinese herbal medicines were dissolved in methanol and added to the culture. Bacterial growth was determined at 600 nm after incubation for 8 h at 37 °C.

### 4.3. Purification of Active Compound

*N. incisum* powder was soaked in ethyl acetate and extracted by ultrasonic waves. After the filtrate was evaporated and dried, the extract was dissolved with methanol. The methanol-soluble extract of *N. incisum* was fractionated via a silica gel gradient vacuum liquid chromatography column eluting with petroleum ether and a step gradient of CH_2_Cl_2_/MeOH (*v*/*v* 100:0, 100:1, 50:1, 20:1, and 0:100) to give 6 fractions, based on TLC analysis. Fraction 4 obtained from CH_2_Cl_2_/MeOH (*v*/*v* = 50:1) was subjected to Sephadex LH-20 chromatography with methanol to afford 29 subfractions (S1–S29). Subfractions were analyzed by TLC, and 3 subfractions (S18–S20) had anti-QS activity. Subfraction S20 was further purified into four constituents by preparative high-performance liquid chromatography (HPLC) (Shimadzu, Kyodo, Japan) (70% MeOH/H_2_O). The anti-QS activities of the four constituents were determined by the PQSI selector.

### 4.4. Growth Curve Assay

The overnight culture of *P. aeruginosa* PAO1 was diluted to an OD600 of 0.01 with fresh LB medium and incubated with falcarindiol (0–50 µM) at 37 °C. Bacterial growth was observed by measuring OD at 600 nm.

### 4.5. Agar Well-Diffusion Assay

The *P. aeruginosa* QSIS-*lasI* biosensor was used to detect the QS inhibitory effects of falcarindiol [29]. Falcarindiol and C30 were dissolved in methanol and added into wells made in LB agar medium mixed with an overnight culture of *P. aeruginosa* QSIS-*lasI*, sucrose, 0.75 mg/mL 2,3,5-triphenyltetrazolium chloride (TTC), and 60 nM 3-oxo-C12-HSL. The red zones around the wells were observed after incubating at 37 °C overnight.

### 4.6. Analysis of lasB, pqsA, and rhlA Transcriptional Activity in E. coli

The detection of β-galactosidase activity in *E. coli* MG4/pKDT17, *E. coli* pEAL08-2 [30,31], and *E. coli* pDSY was described previously. The overnight culture of *E. coli* MG4/pKDT17, *E. coli* pEAL08-2, and *E. coli* pDSY was diluted 1:100 in LB and then incubated with falcarindiol at 37 °C for 8 h. A β-galactosidase Assay Kit (Beyotime, Shanghai, China) was used to quantify β-galactosidase activities.

### 4.7. Virulence Factor Assays

The overnight culture of *P. aeruginosa* PAO1 was diluted 1:100 with fresh LB and then incubated with falcarindiol at 37 °C. After incubation for 6 h, the supernatant was collected for the quantification of virulence factors. Pyocyanin was extracted from 800 μL supernatant with 600 μL chloroform. The 500 µL organic layer was incubated with 200 μL HCl (0.2 N) at 37 °C for 30 min. A 150 µL aliquot of the water layer was removed and its OD520 was measured [32]. Elastase activity was measured through elastin Congo red assays. The 800 μL elastin Congo red solution (3 mg/mL in 0.1 M Tris-HCl pH = 7.2; 1 mM CaCl_2_) was incubated with 400 μL cell-free supernatant at 37 °C for 6 h. The samples were centrifuged and measured at 490 nm [33]. The rhamnolipid was measured by an orcinol assay [34]. An 800 µL aliquot of supernatant was extracted with 1 mL diethyl ether. The organic phase (800 µL) was evaporated, and the residue was resuspended in a solution (100 µL sterile water, 700 µL 70% H_2_SO_4_, and 100 µL 1.6% orcinol). The solution was measured at 420 nm after incubating at 80 °C for 30 min.

### 4.8. Biofilm Formation Assay

The overnight culture of *P. aeruginosa* PAO1 was diluted 1:10,000 with fresh LB and transferred into a 1.5 mL centrifuge tube with falcarindiol at 2.5 μM to 20 μM. Non-adherent cells were eliminated by PBS after static incubation for 12 h. The biofilm was stained with 0.1% crystal violet for 15 min. The stained biofilm was dissolved with 33% (*v*/*v*) glacial acetic acid and measured at OD590 nm.

### 4.9. Real-Time RT-PCR Assay

The overnight *P. aeruginosa* PAO1 culture was diluted 1:100 and then incubated with falcarindiol at 50 μM for another 6 h. Total RNA was isolated with an RNA isolation kit (Nobelab Biotechnologies, Beijing, China) [35]. HiScript III RT Super Mix (Vazyme Biotech, Nanjing, China) and ChamQ Universal SYBR qPCR Master Mix (Vazyme Biotech, Nanjing, China) were used for reverse transcription and amplification, respectively. The reference gene *rpsl* was used to normalize qRT-PCR. The comparative threshold method (2^−∆∆Ct^) was used to calculate the relative expression of the target genes.

### 4.10. EMSA

The recombinant expression vector pET28a-LasR (for LasR production) was transformed into *E. coli* BL21 (DE3). The transformants were incubated with 1 mM IPTG and 100 μM 3-oxo-C12-HSL at 18 °C for 16 h. Whole-cell lysates containing the LasR protein were collected and used in the EMSA [36]. The promoter of the *lasI* gene was amplified from the genome of *P. aeruginosa* and used as a probe. Biotin-labeled P*lasI* probe was prepared by the EMSA probe labeling kit (Beyotime, Shanghai, China). Different concentrations of falcarindiol (0 μM, 12.5 μM, 25 μM, 50 μM, 75 μM, and 100 μM, dissolved in DMSO) were added to the reaction mix. The binding reactions for LasR-P*lasI* interaction and gel-shift assays were performed with the EMSA/Gel-Shift Kit (Beyotime, Shanghai, China).

### 4.11. Molecular Docking Analysis

Molecular docking was performed as previously reported [24]. The structure of the receptor protein LasR (PDB ID, 3IX3) was downloaded from the Protein Data Bank (PDB). The molecular docking study was performed by Autodock 4.2.6 (The Scripps Research Institute, Jolla, LA USA). The conformations and interactions between falcarindiol and LasR were visualized by PyMol 2.3.0 (Schrodinger, New York, NY, USA). and LigPlot+ v.1.4 (EMBL-EBI, Cambridge, UK).

## 5. Conclusions

Our results indicate that falcarindiol identified from *N. incisum* can inhibit the QS of *P. aeruginosa* and exert a significant inhibitory effect on the formation of biofilm and the production of virulence factors (elastase, pyocyanin, and rhamnolipid). The mRNA expression of QS-related genes (*lasB*, *phzH*, *rhlA*, *lasI*, *rhlI*, *pqsA*, and *rhlR*) was downregulated by falcarindiol. Falcarindiol inhibited the binding of the transcription factor LasR with the *lasI* promoter region and the transcriptional activation of downstream genes. Falcarindiol has great potential to be developed as an anti-infective agent. The therapeutic effects of falcarindiol on *P. aeruginosa* infection need to be further studied.

## Supplementary Materials

The following are available online. Table S1: Bacterial strains and plasmids. Table S2: The primer sequences for cloning. Table S3: The primer sequences for real-time RT-PCR. Table S4: NMR spectroscopy of the active compound purified from the *Notopterygium incisum* extract. Table S5: Details of the docked complex of LasR with 3-oxo-C12-HSL and falcarindiol. Figure S1: The inhibitory activity of Chinese herbal medicines against PQSI selector. Figure S2: The inhibitory activity of *Notopterygium incisum* extract against PQSI selector. Figure S3: HPLC analysis of the active compound purified from the *Notopterygium incisum* extract. Figure S4: ^1^H-NMR spectroscopy of the active compound purified from the *Notopterygium incisum* extract (Solvent: DMSO-d6). Figure S5: ^13^C-NMR spectroscopy of the active compound purified from the *Notopterygium incisum* extract (Solvent: DMSO-d6). Figure S6: Comparison of HPLC retention times of the active compound purified from the *Notopterygium incisum* extract (A), the chemical reference substance of falcarindiol (B), and their mixture (C).

## Sample Availability

Samples of the compounds are available from the authors.
