# Supplementary material for: Falcarindiol Isolated from Notopterygium incisum Inhibits the Quorum Sensing of Pseudomonas aeruginosa
Source: Molecules. 2021 Sep 29;26(19):5896. doi: 10.3390/molecules26195896 (PMC8512080; doi:10.3390/molecules26195896)
Supplement: Supplementary file 1 [file molecules-26-05896-s001.zip › molecules-1327699-supplementary.pdf]

Falcarindiol isolated from *Notopterygium incisum* inhibits the quorum sensing of *Pseudomonas aeruginosa*

Chaoyue Zhao [abcd,1] , Hongda Zheng [abcd,1], Liman Zhou[abcd], Hongrui Ji [abcd], Lu Zhao [abcd], Wengong Yu [abcd] and Qianhong Gong [abcd,*]

[a] School of Medicine and Pharmacy, Ocean University of China, China

[b] Laboratory for Marine Drugs and Bioproducts, Qingdao National Laboratory for Marine Science and Technology, China

[c] Provincial Key Laboratory of Glycoscience and Glycotechnology, Ocean University of China, China

[d] Key Laboratory of Marine Drugs, Chinese Ministry of Education, School of Medicine and Pharmacy, Ocean University of China, China

Table S1. Bacterial strains and plasmids

Strain or plasmid	Relevant genotype	Source
**Strains**		
P. aeruginosa PAO1	Wild type	Schuster and Greenberg. (2007)
PQSI	Wild type PAO1 harboring plasmid pPqsA-SacB	This study
P. aeruginosa PAO-MW1	D*lasI*::Tet D*rhlI*::Tn501-2 strain PAO1 derivative	Wang et al. (2011)
QSIS-*lasI*	PAO-MW1 harboring plasmid pMHLASI	
E. coli MG4/pKDT17	*E. coli* DH5a harboring plasmid MG4/pKDT17	Hong et al. (2012)
E. coli pEAL08-2	*E. coli* DH5a harboring plasmid pEAL08-2	Cugini et al. (2007)
E. coli pDSY	*E. coli* DH5a harboring plasmid pDSY	This study
E. coli LasR	*E. coli* BL21 harboring plasmid pET28a-LasR	This study
**Plasmids**		
pLasB-SacB1	P*lasB*-*sacB* transcriptional fusion	Rasmussen et al. (2007)
pPqsA-SacB	P*pqsA*-*sacB* transcriptional fusion	This study
pMHLASI	P*lasI-sacB,* and P*lac*-*lasR* transcriptional fusion	Wang et al. (2011)
MG4/pKDT17	P*lasB*-*lacZ*, and P*lac*-*lasR* transcriptional fusion	Pearson et al. (1994)
pEAL08-2	P*pqsA*-*lacZ*, and P*lac*-*pqsR* transcriptional fusion	Cugini et al. (2007)
pDSY	P*rhlA*-*lacZ*, and P*lac*-*rhlR* transcriptional fusion	This study
pET28a-LasR	pET-28a (+) vector carrying *lasR* gene from *P. aeruginosa* PAO1 strain	This study

Table S2. The primer sequences for cloning

Primers	sequences
P*pqsA* (F)	GCTCTAGACGAGCAAAGTGGGTTGTCATT
P*pqsA* (R)	ACATGCATGCGATCAGGAGATACCTGAGCCA
pET-28a-LasR (F)	CATATGGCTAGCATGACTGGTGGACAG
pET-28a-LasR (R)	CTCGAGTGCGGCCGCAAGCTTGAGAG
P*lasI* (F)	CTTCGAGCCTAGCAAGGG
P*lasI* (R)	CTTCCTCCAAATAGGAAGCT

Table S3. The primer sequences for real-time RT-PCR

Primers	sequences	primers	sequences
rpsls (F)	GCAACTATCAACCAGCTGGTG	*rpsls* (R)	GCTGTGCTCTTGCAGGTTGTG
lasR (F)	CTGTGGATGCTCAAGGACTAC	*lasR* (R)	AACTGGTCTTGCCGATGG
lasI (F)	GGCTGGGACGTTAGTGTCAT	*lasI* (R)	AAAACCTGGGCTTCAGGAGT
lasB (F)	ACCAGAAGATCGGCAAGTAC	*lasB* (R)	GTTGACCTGCTTGTAGGTGTTG
rhlR (F)	CTGGGCTTCGATTACTACGC	*rhlR* (R)	CCCGTAGTTCTGCATCTGGT
rhlI (F)	GTAGCGGGTTTGCGGATG	*rhlI* (R)	CGGCATCAGGTCTTCATCG
rhlA (F)	GGCGATCGGCCATCT	*rhlA* (R)	AGCGAAGCCATGTGCTGAT
pqsR (F)	CTGATCTGCCGGTAATTGG	*pqsR* (R)	ATCGACGAGGAACTGAAGA
pqsA (F)	GACCGGCTGTATTCGATTC	*pqsA* (R)	GCTGAACCAGGGAAAGAAC
phzH (F)	TGCGCGAGT TCAGCCACCTG	*phzH* (R)	TCCGGGACATAGTCGGCGCA

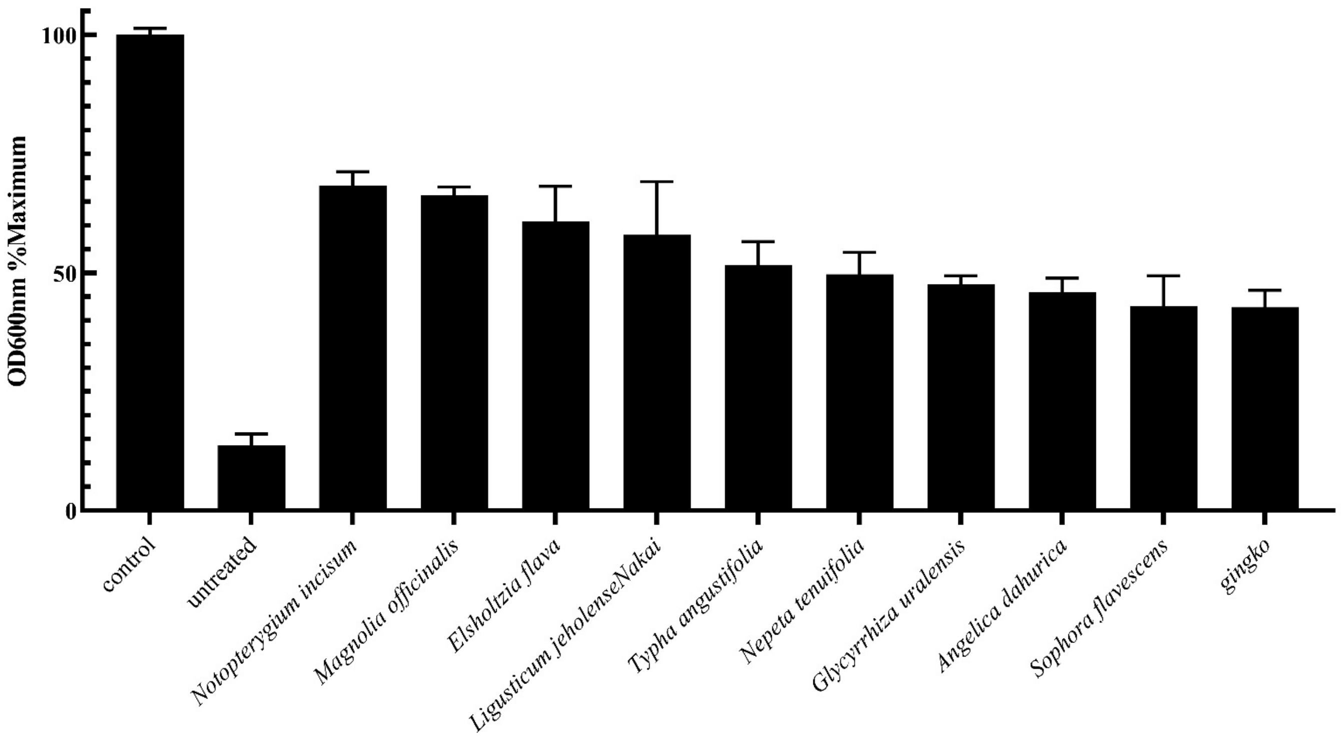

Figure. S1. The inhibitory activity of Chinese herbal medicines against PQSI selector

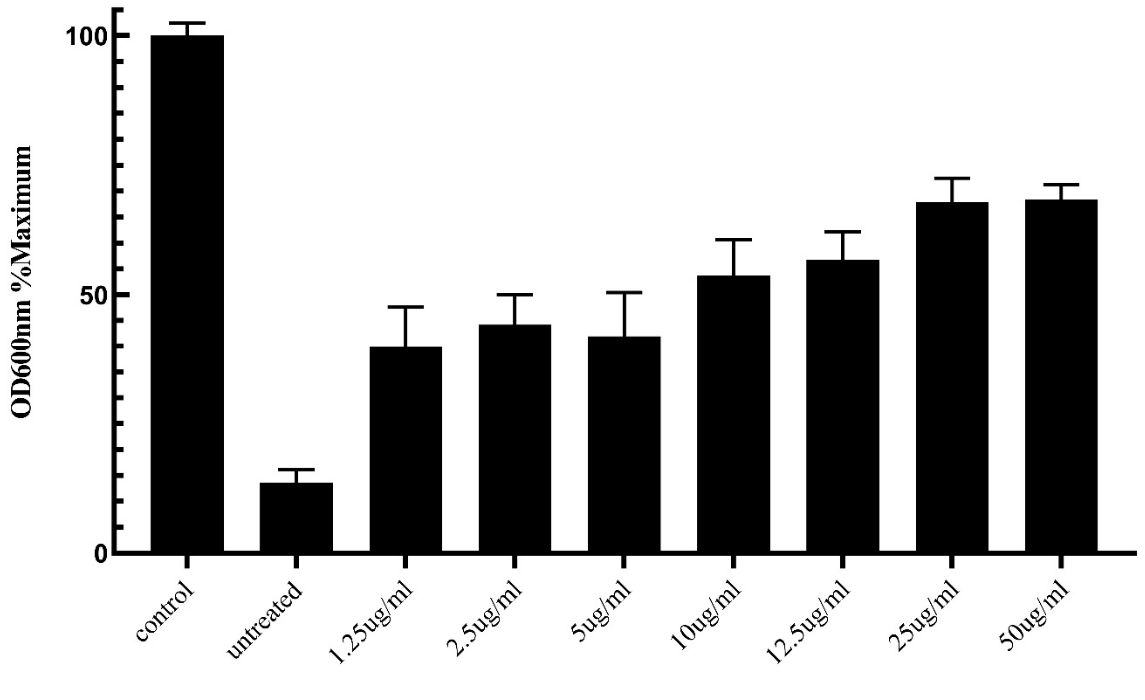

Figure. S2. The inhibitory activity of *Notopterygium incisum* extract against PQSI selector

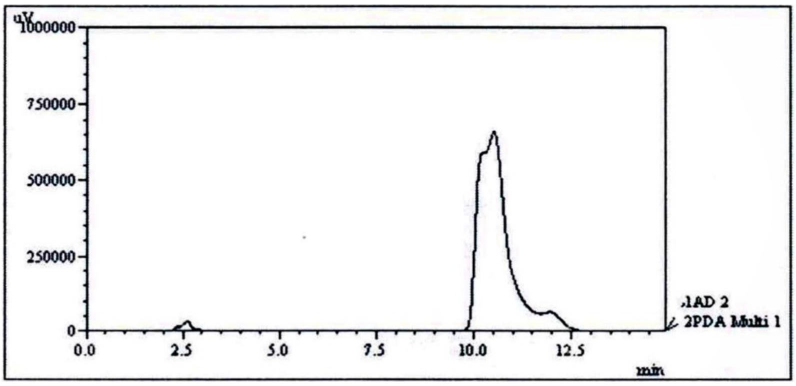

Figure. S3. HPLC analysis of the active compound purified from the *Notopterygium incisum* extract

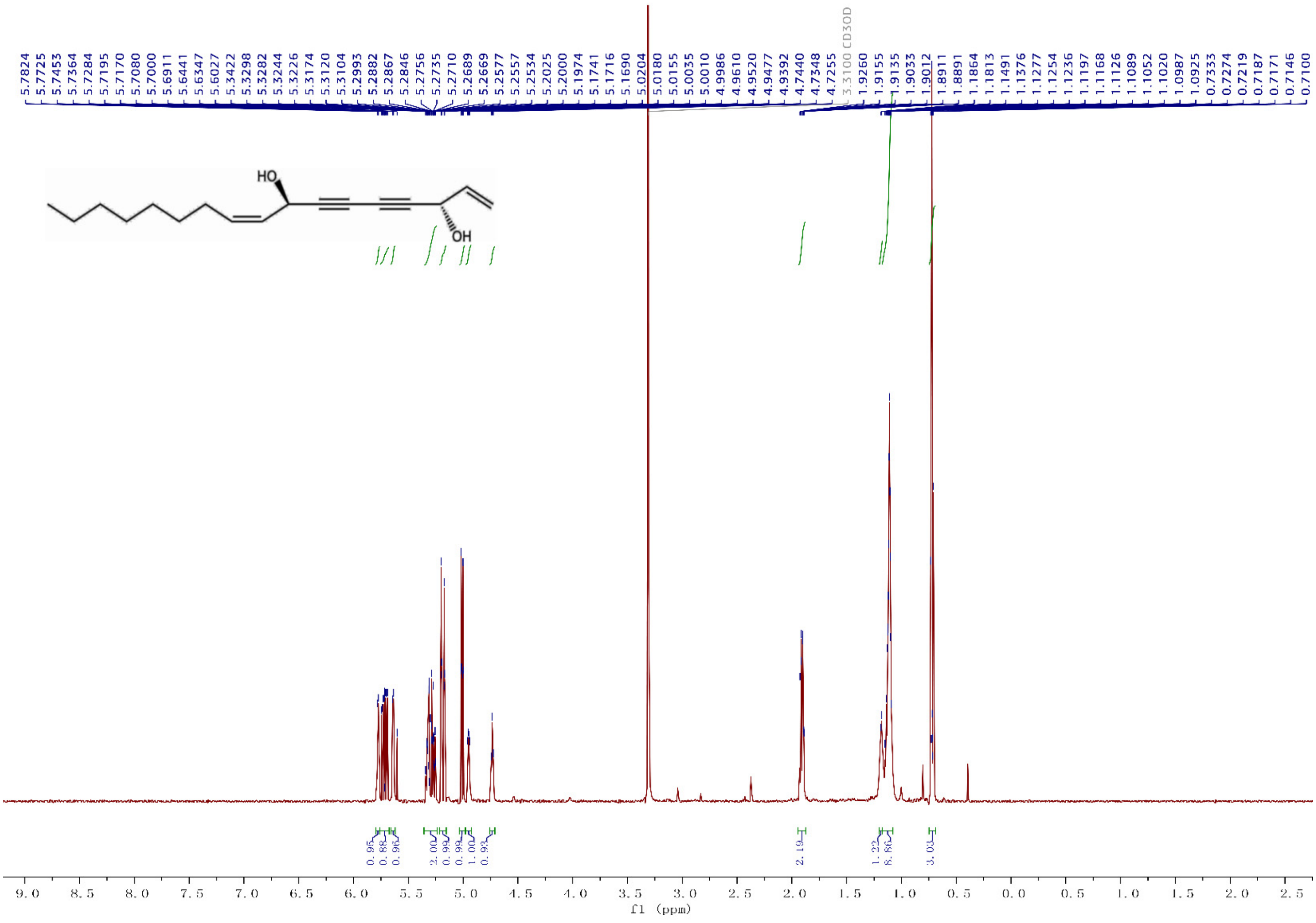

Figure. S4. ^{1}H-NMR spectroscopy of the active compound purified from the *Notopterygium incisum* extract (Solvent: DMSO-d6)

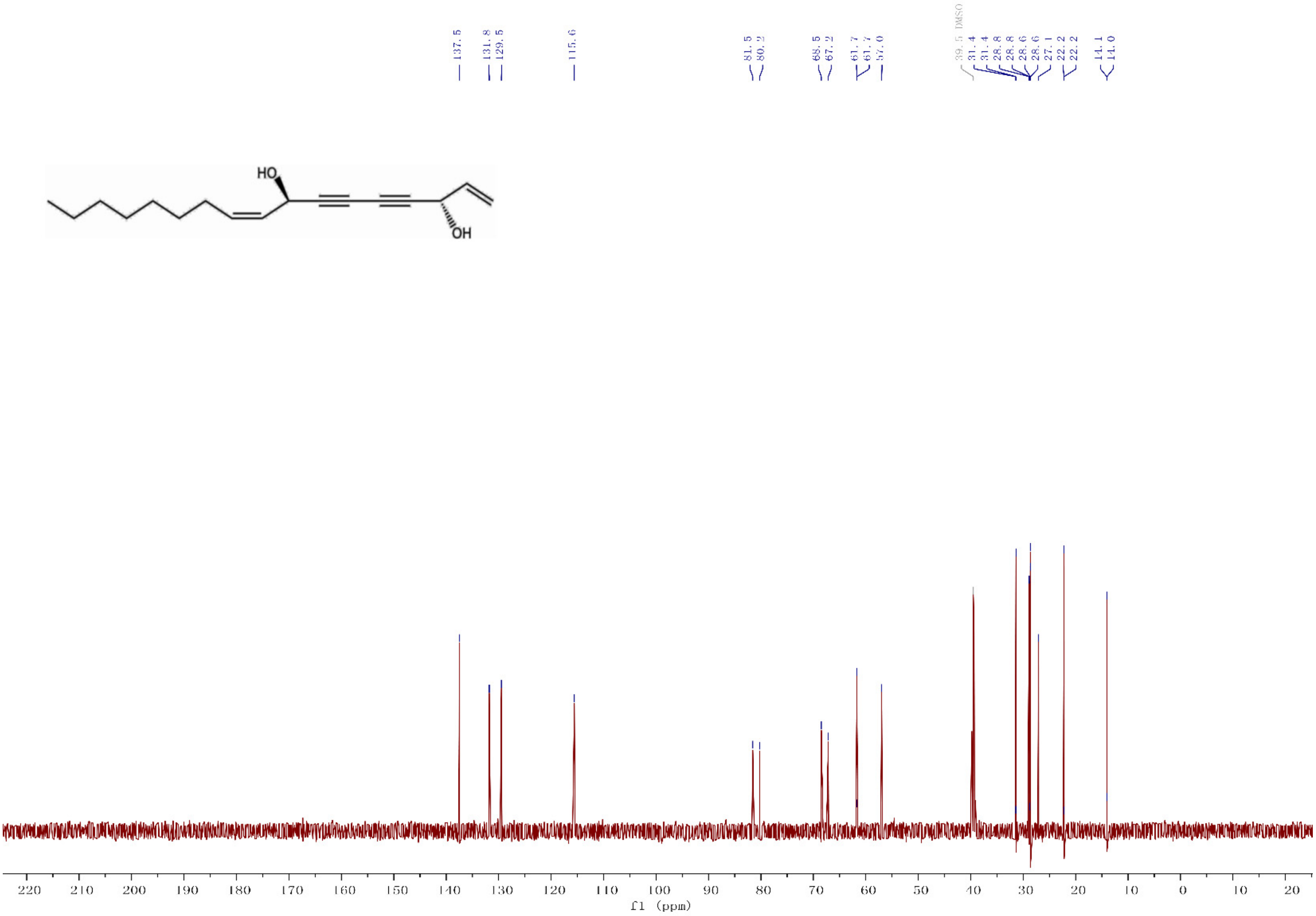

Figure. S5. ^{13}C-NMR spectroscopy of the active compound purified from the *Notopterygium incisum* extract

(Solvent: DMSO-d6)

The relative molecular mass of the active compound from *Notopterygium incisum* extract was 260.2, which was expressed as ESI-MS m/z: 243.2 [M+1-H_2O], 225.2 [M+1-2 H_2O].

^{1}H NMR (600 MHz, DMSO-d_6) δ 5.78 (d, J = 5.9 Hz, 1H), 5.76 – 5.68 (m, 1H), 5.64 (d, J = 5.6 Hz, 1H), 5.36 – 5.24 (m, 2H), 5.19 (dt, J = 17.1, 1.5 Hz, 1H), 5.01 (dt, J = 10.1, 1.5 Hz, 1H), 4.95 (dd, J = 7.8, 5.2 Hz, 1H), 4.73 (t, J = 5.6 Hz, 1H), 1.94 – 1.87 (m, 2H), 1.20 – 1.18 (m, 1H), 1.18 – 1.08 (m, 9H), 0.75 – 0.69 (m, 3H). ^{13}C NMR (151 MHz, DMSO-d_6) δ 137.48, 131.79, 129.50, 115.57, 81.55, 80.23, 68.48, 67.16, 61.71, 56.99, 31.38, 28.84, 28.64, 28.60, 27.06, 22.19, 14.03.

Table S4. NMR spectroscopy of the active compound purified from the *Notopterygium incisum* extract

	falcarindiol	
Position	δC	δH(m, J Hz)
1	116.0, CH2	5.30, dt
		5.13, dt
2	138.0, C	5.85, ddd
3	62.2, CH	4.86, dd
4	82.0, C	
5	69.0, C	
6	67.6, C	
7	80.7, C	
8	57.5, CH	5.08, dd
9	130.0, CH	5.40, m
10	132.3, CH	5.45, m
11	31.9, CH2	2.03, m
12	29.3, CH2	1.30, m
13	29.1, CH2	1.27, m
14	29.1, CH2	1.26, m
15	27.5, CH2	1.24, m
16	22.7, CH2	1.24, m
17	14.5, CH3	0.85, t
3-OH		5.90, br d
8-OH		5.77, br d

600MHz, DMSO-d6 (2.50 ppm). 150MHz, DMSO-d6 (39.5 ppm)

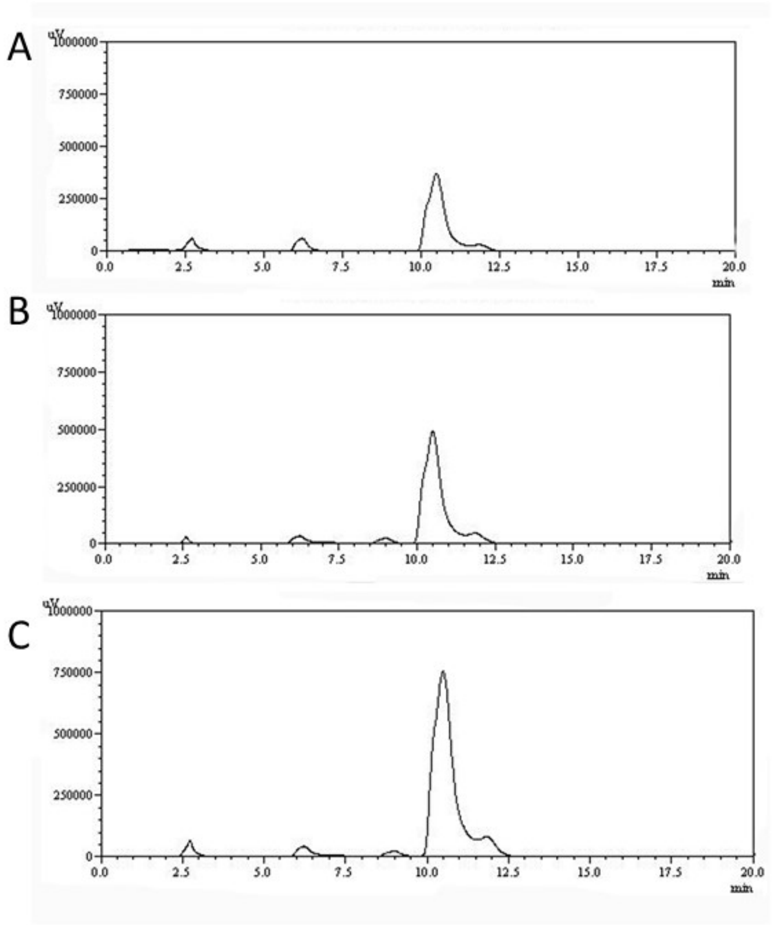

Figure. S6. Comparison of HPLC retention times of the active compound purified from the *Notopterygium incisum* extract (A), the chemical reference substance of falcarindiol (B) and their mixture (C)

Table S5. Details of the docked complex of the LasR with 3-oxo-C12-HSL and falcarindiol

**Molecule**	**affinity (kcal/mol)**	**Hydrogen bonding interactions**
3-oxo-C12-HSL	-8.0	Ser129, Tyr56, Asp73, Trp60, Thr75
falcarindiol	-7.5	Tyr47, Ser129, Thr75
